# Biomechanical study on the treatment of tibial defects in total knee arthroplasty using the cement-screw and metal block with extension stem techniques: a finite element analysis

**DOI:** 10.3389/fbioe.2026.1721687

**Published:** 2026-02-04

**Authors:** Yafei Lu, Zhongjian Tang, Qiang Gu, Zhexi Zhu, Wenrui Liu, Ziqiang Zhu, Gang Duan

**Affiliations:** 1 Department of Orthopaedics, The Second Affiliated Hospital of XuZhou Medical University, Xuzhou, China; 2 Graduate School of Xuzhou Medical University, Xuzhou, China; 3 Department of Trauma Orthopaedics, Peking University People’s Hospital, Beijing, China; 4 Graduate School, Hebei Medical University, Hebei, China

**Keywords:** bone defects, finite element analysis, the cement-screw technique, the metal block extension stem technique, total knee arthroplasty

## Abstract

**Objective:**

To compare the biomechanical properties of the cement-screw technique with the metal block extension stem technique in reconstructing Anderson Orthopaedic Research Institute (AORI) type 2 tibial defects in total knee arthroplasty using the finite element method, in order to provide a basis for clinical selection.

**Methods:**

Non-inclusive 5-mm and 10-mm depth AORI type 2 tibial defects were modeled using the finite element method. The cement-screw technique and the metal block extension stem technique were applied for reconstruction, resulting in a total of four sets of finite element models. Each group of models was tested under two loads: horizontal walking and descending stairs. The von Mises stress distributions in the tibia, prosthesis, and bone defect regions, as well as the peaks of micromotion at the prosthesis-tibia interface, were measured.

**Results:**

In the AORI type 2 tibial defect model, the cement-screw group, when reconstructing 5 mm and 10 mm defects under horizontal walking and descending stairs loads, exhibited higher maximum tibial stress (5 mm: 78.0–140 MPa; 10 mm: 80.9–151 MPa), proximal tibial defect area stress (5 mm: 11.3–25.3 MPa; 10 mm: 10.8–24.1 MPa), and peak micromotion values (5 mm: 9.90–26.99 μm; 10 mm: 11.94–31.98 μm) compared to the metal block extension stem group (tibial stress 5 mm: 73.2–130 MPa, 10 mm: 66.6–118 MPa; defect area stress 5 mm: 7.83–16.3 MPa, 10 mm: 8.54–18.8 MPa; peak micromotion 5 mm: 6.56–14.72 μm, 10 mm: 8.92–24.09 μm). However, prosthesis stresses were lower in the cement-screw group (5 mm: 87.1–183 MPa; 10 mm: 60.2–158 MPa) than in the metal block extension stem group (5 mm: 101–194 MPa, 10 mm: 92.7–167 MPa). Under horizontal walking loading, the two techniques showed no superiority of one over the other in terms of the von Mises stresses and the peaks of micromotion; however, under descending stairs loads, the maximum stress in the tibia of the cement-screw group with a 10-mm defect exceeded 150 MPa (151 MPa), indicating a potential fracture risk, and the peaks of micromotion was smaller in all models.

**Conclusion:**

The findings of this study indicate that the cement-screw technique is more cost-effective and convenient for repairing 5-mm defects and is appropriate for reconstruction of this size. However, when the bone defect reaches 10 mm, the cement-screw technique may elevate the risk of fracture, and thus, safety concerns must be taken into account. In contrast, the metal block extension stem technique offers a better balance between effectiveness and safety, making it the preferred option for defects of this size.

## 1 Introduction

Osteoarthritis (OA) is a common degenerative joint disease, with the knee joint being one of the most frequently affected joints. The prevalence of knee OA approaches 30% among individuals aged 45 and above, and it shows an upward trend with the aging population (Katz et al., 2021; Gelber, 2024; Sharma, 2021). Total knee arthroplasty (TKA) is the standard surgical procedure for treating end-stage knee osteoarthritis, alleviating pain, restoring joint function, and correcting deformities (Martina et al., 2022; Tampere et al., 2025; Liu et al., 2023). However, severe OA and revision surgeries are often accompanied by bone defects. The stable reconstruction of these defects directly influences the fixation effectiveness and long-term survival rate of the prosthesis, thereby determining the patient’s postoperative functional recovery (Alasaad and Ibrahim, 2023; Kayani et al., 2024).

Currently, there are various repair methods for tibial plateau bone defects, including simple cement filling, cement-screw fixation, metal block, autologous or allograft bone grafting, and adequate osteotomy combined with thick polyethylene inserts (Huten et al., 2021; Özcan et al., 2021; Sculco et al., 2016; Chon et al., 2021; Garceau et al., 2020). The repair strategy should be individually selected based on the type of bone defect and paired with an appropriate prosthetic implant combination to achieve optimal clinical outcomes. Among the various bone defect classification systems, the Anderson Orthopaedic Research Institute (AORI) classification is the most widely used, categorizing tibial defects into three types based on their size and location (Rodríguez-Merchán et al., 2021). However, for AORI type 2 bone defects, there is currently no unified consensus on reconstruction. Clinically, two main techniques are employed: one is the cement-screw technique (such as the Attune knee system) (Harna et al., 2025), and the other is the metal block extension stem technique (such as the ACCK knee system) (Bieganowski et al., 2022).

The cement-screw technique has garnered significant attention due to its simplicity, short surgical time, low cost, and ability to maximize bone preservation, along with good dimensional adaptability. However, this technique may encounter issues such as cement compression, fracture, and even prosthetic loosening when dealing with larger non-contained defects. Additionally, the exothermic effect during cement curing may lead to necrosis of the surrounding bone tissue (Harna et al., 2025; Zheng et al., 2020). In contrast, the metal block technique offers higher stiffness and faster postoperative recovery but is expensive and often requires the removal of more healthy bone tissue to match the shape of the metal block (Arab et al., 2020). Currently, these evaluations are mostly based on clinical indicators, and a systematic comparison of the biomechanical characteristics of the two methods is still lacking.

Therefore, this study aims to compare the biomechanical performance differences between the cement-screw technique and the metal block extension stem technique in reconstructing AORI type 2 tibial defects through finite element analysis (FEA), with a focus on evaluating stress distribution and stability characteristics. Finite element analysis, as a computer simulation technology based on mathematical approximation methods, can accurately reproduce the geometric morphology and mechanical environment of bone structures and has been widely applied in orthopedic biomechanical research. It offers advantages such as flexible model construction, ease of operation, and reliable results (Apostolopoulos et al., 2024). This study hopes to provide theoretical evidence and practical guidance for clinical selection of reconstruction strategies.

## 2 Materials and methods

### 2.1 3D modeling and surgical simulation of the Tibia and implants

The studies involving human participants were reviewed and approved by the Ethics Committee of the Second Affiliated Hospital of Xuzhou Medical University. Written informed consent to participate in this study was provided by the participant. Written informed consent was obtained from the individual for the publication of any potentially identifiable images or data included in this article. All procedures strictly adhered to the ethical guidelines outlined in the Declaration of Helsinki. The research subject was a 68-year-old male volunteer recruited from the Department of Orthopedics at the Second Affiliated Hospital of Xuzhou Medical University. The volunteer’s preoperative dual-energy X-ray absorptiometry (DXA) scan revealed normal bone mineral density (T-score > −1.0), with no evidence of osteoporosis. He was 175 cm tall, weighed 81 kg, and was diagnosed with left knee osteoarthritis. A Siemens 128-slice spiral CT scanner was employed to perform thin-slice scans of the volunteer’s left tibia, obtaining high-resolution and high-precision imaging data. The raw image data obtained was stored in DICOM format.

The image data was imported into Mimics 21.0 software, where threshold segmentation, region growing, and other steps were carried out to establish a tibial model, which was then exported in STL format. Subsequently, the model was imported into Geomagic Studio 2021 software for mesh refinement, surface smoothing, contour line extraction, and other processing. Using the offset function, the model was offset inward, and the resulting inner model was defined as the cancellous bone model. This model was then imported into Solidworks software, where Boolean operations were utilized to construct a three-dimensional model of both cortical and cancellous bones, ultimately building a 3D tibial model. The implant data obtained from a 3D scanner was imported into Geomagic Design X 2022 software for reverse modeling to generate an implant model.

**FIGURE 1:**
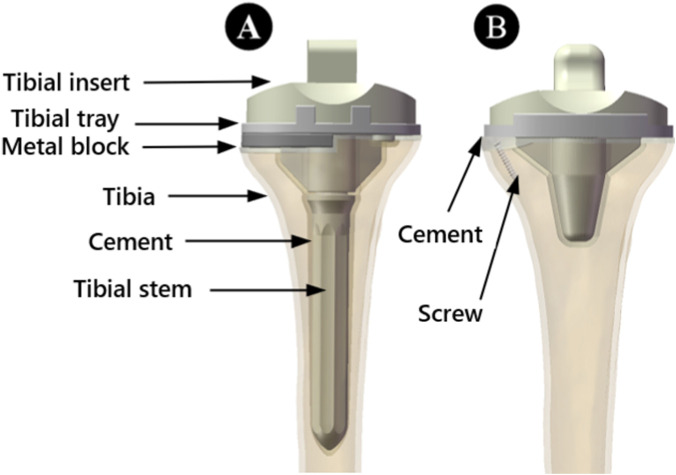
Frontal view of the assembled model of a 5 mm bone defect. **(A)** Treatment using the metal block extension stem technique. **(B)** Treatment using the cement-screw technique.

**FIGURE 2:**
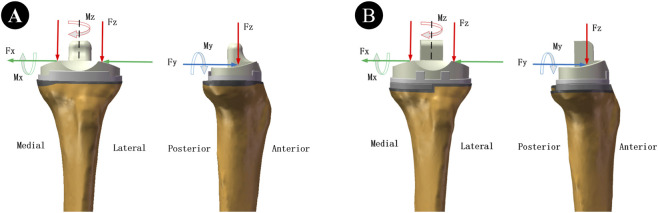
Schematic diagram of horizontal walking and descending stairs loads. **(A)** Treatment using the cement-screw technique. **(B)** Treatment using the metal block extension stem technique. (Fx, Fy, and Fz represent the medial-lateral, anterior-posterior and axial tibio-femoral contact forces, respectively. Mx, My, and Mz denote the flexion-extension, inversion-eversion moment and internal-external rotation moment respectively).

Following standard surgical procedures and under the guidance of an experienced surgeon, GD, surgical simulations were conducted. First, osteotomy was performed 8 mm below the articular surface of the medial tibial plateau with a posterior slope of 6°. Based on previous research, we selected wedge-shaped defects (Figures 1, 2) to conduct additional resection of the medial plateau, thus constructing non - enclosing tibial medial bone defect models with depths of 5 mm and 10 mm. The cement-screw technique and metal block extension stem technique were then employed to reconstruct the bone defects.

In the cement-screw technique, we employed two screws (3.5 mm diameter, 20 mm length) as per previous literature, inserting them in a typical anteroposterior direction. The screw heads were positioned slightly below the tibial plateau, and bone cement was used to secure the prosthesis implant to the bone (Harna et al., 2025; Shao et al., 2025). The metal block and the tibial plateau were fixed with screws. The relevant implants for both techniques were sequentially assembled onto the bone defect models (Figure 1).

### 2.2 Material properties

In this study, materials such as bone and prosthetic implants are defined as continuous, homogeneous, isotropic linear elastic materials. Although actual bone (particularly cortical bone) exhibits anisotropy, a substantial body of published finite - element studies on the knee joint indicates that stress distributions, peak stresses, and relative comparative trends obtained under the isotropic assumption are effective and reliable for evaluating the biomechanical performance of different implant designs (Arab et al., 2020; Apostolopoulos et al., 2024; Shao et al., 2025). Ti6Al4V was used as the material for the tibial prosthesis implant, and UHMWPE served as the Tibial insert material. According to previous studies (Sculco et al., 2016; Chon et al., 2021; Garceau et al.; Rodríguez-Merchán et al., 2021; Harna et al., 2025; Bieganowski et al., 2022; Zheng et al., 2020; Arab et al., 2020; Apostolopoulos et al., 2024; Shao et al., 2025; Liu et al., 2020; Wan et al., 2024; Liu et al., 2025), we established the modulus of elasticity for cortical bone and cancellous bone at 14.0 GPa and 0.7 GPa, respectively, and set the Poisson’s ratio for both at 0.30. For the prosthetic implant, we assigned a modulus of elasticity of 110 GPa and a Poisson’s ratio of 0.3. The material parameters for these components are detailed in Table 1. It is worth noting that this finite element model of the tibia has been validated in a previous study (Liu et al., 2025).

**TABLE 1:** Material parameters of the finite element model.

Component	Elastic modulus (MPa)	Poisson’s ratio
Cortical bone	14,000.0	0.30
Cancellous bone	700.0	0.30
Tibial insert	500.0	0.40
Tibial tray and stem	110,000.0	0.30
Metal block	110,000.0	0.30
Cement	3,000.0	0.37
Screw	110,000.0	0.30

### 2.3 Boundary conditions and loading settings

In this finite element analysis, The insert-tray, tray-cement, stem-cement, cement-bone, and cement-screw interfaces were set as tied contacts. The remaining interfaces were set to sticky connection (an enforced condition during which contact interfaces will not enter the sliding phase) (Liu et al., 2020). Finite element analysis was conducted using ANSYS Workbench 2021 R2 software. The applied loads and methods were based on previous studies (Kutzner et al., 2010; Xie et al., 2020). The loads selected for this study were horizontal walking and descending stairs. According to the pre-experiment, we selected the force and moment corresponding to the resultant peak point in the motion cycle as the applied loads (Table 2). The distal tibia was set to be fully restrained, and the forces and moments for the loading scenarios are shown (Figure 2).

**TABLE 2:** Parameters of applied loads.

Type of activity	Horizontal walking	Descending stairs
Mx (N*mm)	−10650	−24280
My (N*mm)	−14800	1,120
Mz (N*mm)	−1,146	−7,000
Fx(N)	30	−70
Fy(N)	121	210
Fz(N)	−1,348	−2,450

### 2.4 Model validation

To ensure the accuracy of the study, we constructed a complete tibial defect model as well as two different implant combination models, and assigned corresponding material properties to these models based on the methods described in references (Liu et al., 2020; Wan et al., 2024; Liu et al., 2025). Subsequently, we fully constrained the degrees of freedom at the distal end of the tibial defect model and applied forces simulating horizontal walking on the tibial plateau: Fx = 30 N, Fy = 121 N, and Fz = −1348 N; as well as forces simulating descending stairs: Fx = −70 N, Fy = 210 N, and Fz = −2450 N (where Fx, Fy, and Fz represent the medial-lateral, anterior-posterior, and axial tibiofemoral contact forces, respectively; the negative sign indicates the opposite direction). Using ANSYS Workbench 2021 R2 software, we conducted an in-depth analysis of these models and meticulously compared the obtained results with the reported data in references (Liu et al., 2020; Liu et al., 2025), thereby validating the effectiveness of the constructed models.

### 2.5 Key evaluation metrics

The present study is based on Finite Element Analysis (FEA), and the primary output parameters include the distribution characteristics of the maximum von Mises stress (VMS) and the peaks of micromotion. In particular, the maximum von Mises stress should be utilized to evaluate the risk of fracture and prosthesis breakage, while the peaks of micromotion should be used to assess the stability and the tendency for loosening of the prosthesis implant. The micromotion value is calculated using a custom code that computes the relative displacement between a node on the prosthesis and its nearest bone tissue node.

## 3 Results

### 3.1 von Mises stress distribution and the peaks of micromotion during horizontal walking

#### 3.1.1 von Mises stress (VMS) distribution and results for the tibia

**FIGURE 3:**
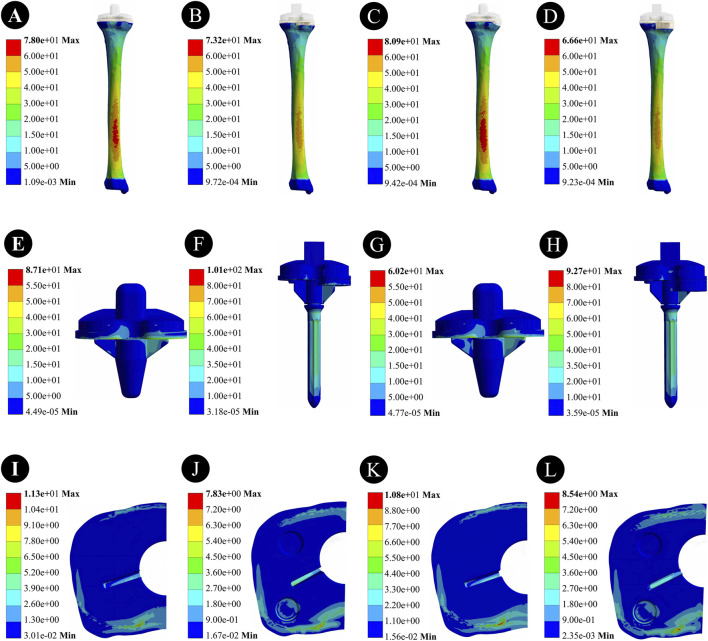
Stress distribution results of two prosthetic implants with different defect depths under horizontal walking load. **(A,E,I)** Stress distribution in the tibia, prosthesis, and proximal tibial bone defect region in the cement-screw group at a 5 mm bone defect depth; **(B,F,J)** Stress distribution in the tibia, prosthesis, and proximal tibial bone defect region in the metal block extension stem group at a 5 mm bone defect depth; **(C,G,K)** Stress distribution in the tibia, prosthesis, and proximal tibial bone defect region in the cement-screw group at a 10 mm bone defect depth; **(D,H,L)** Stress distribution in the tibia, prosthesis, and proximal tibial bone defect region in the metal block extension stem group at a 10 mm bone defect depth.

For a 5-mm bone defect, the maximum stress on the tibia was 78.0 MPa for the cement-screw group and 73.2 MPa for the metal block extension stem group. For a 10-mm bone defect, the maximum stress on the tibia was 80.9 MPa for the cement-screw group and 66.6 MPa for the metal block extension stem group. The maximum stress in the cement-screw group was located on the dorsal side of the tibia, and similarly, in the metal block extension stem group, the maximum stress was also on the dorsal side of the tibia, although the area of high stress was smaller (Figure 3).

#### 3.1.2 von Mises stress (VMS) distribution and results for prostheses

For a 5-mm bone defect, the maximum stress in the cement-screw group was 87.1 MPa, whereas the maximum stress in the metal block extension stem group was 101 MPa, which was 13.8% higher. In the case of a 10-mm bone defect, the maximum stress in the cement-screw group was 60.2 MPa, and the stress in the metal block extension stem group increased by 54.0%–92.7 MPa. The maximum stress point in the cement-screw group was located at the bottom of the tibial plateau, whereas in the metal block extension stem group, the maximum stress point was at the distal end of the extension stem (Figure 3).

#### 3.1.3 von Mises stress (VMS) distribution and results in the proximal tibial bone defect region

For a 5-mm bone defect, the maximum stress in the cement-screw group was 11.3 MPa, whereas the maximum stress in the metal block extension stem group was 7.83 MPa, representing a 30.7% reduction. In the case of a 10-mm bone defect, the maximum stress in the cement-screw group was 10.8 MPa, and the maximum stress in the metal block extension stem group was 8.54 MPa, indicating a 20.9% reduction. There was a significant decrease in stress in the area of the proximal tibial bone defect for the metal block extension stem group (Figure 3).

#### 3.1.4 The peaks of micromotion between the prosthesis and the Tibia

For 5-mm bone defects, the peaks of micromotion was 9.90 μm in the cement-screw group and 6.56 μm in the metal block extension stem group. For 10-mm bone defects, the peaks of micromotion was 11.94 μm in the cement-screw group and 8.92 μm in the metal block extension stem group (Figure 5).

### 3.2 von Mises Stress distribution and the peaks of micromotion during descending stairs

#### 3.2.1 von Mises stress (VMS) distribution and results for the Tibia

**FIGURE 4:**
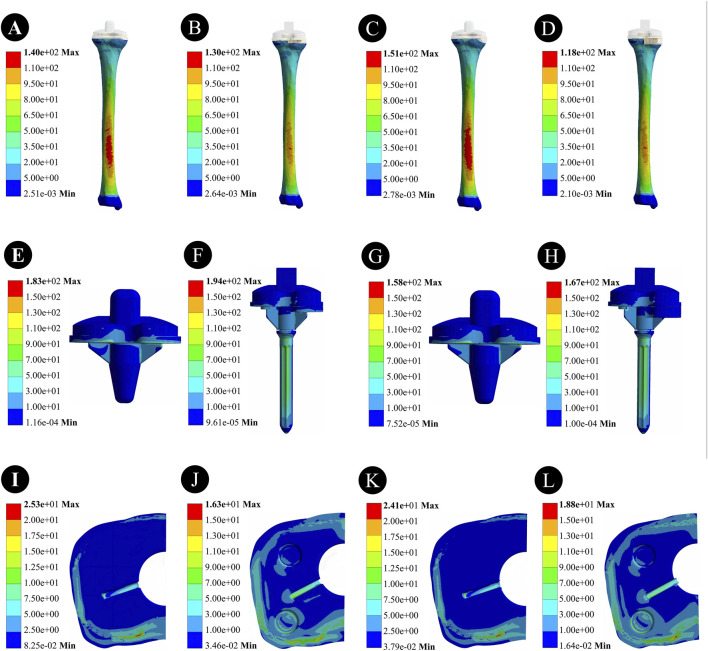
Stress distribution results of two prosthetic implants with different defect depths under descending stairs load. **(A,E,I)** Stress distribution in the tibia, prosthesis, and proximal tibial bone defect region in the cement-screw group at a 5 mm bone defect depth; **(B,F,J)** Stress distribution in the tibia, prosthesis, and proximal tibial bone defect region in the metal block extension stem group at a 5 mm bone defect depth; **(C,G,K)** Stress distribution in the tibia, prosthesis, and proximal tibial bone defect region in the cement-screw group at a 10 mm bone defect depth; **(D,H,L)** Stress distribution in the tibia, prosthesis, and proximal tibial bone defect region in the metal block extension stem group at a 10 mm bone defect depth.

For a 5-mm bone defect, the maximum stress on the tibia was 140 MPa for the cement-screw group and 130 MPa for the metal block extension stem group. For a 10-mm bone defect, the maximum stress on the tibia was 151 MPa for the cement-screw group and 118 MPa for the metal block extension stem group. The maximum stresses for the cement-screw group were located on the dorsal side of the tibia, whereas for the metal block extension stem group, the maximum stress was also on the dorsal side of the tibia, but the area of high stress was much smaller (Figures 4).

#### 3.2.2 von Mises stress (VMS) distribution and results for prostheses

In the case of a 5 mm bone defect, the maximum stress within the cement-screw group was 183 MPa, whereas the maximum stress within the metal block extension stem group was 194 MPa, an increase of 6.01%. For a 10-mm bone defect, the maximum stress within the cement-screw group was 158 MPa; the stress within the metal block extension stem group rose by 5.70%–167 MPa. The point of maximum stress in the cement-screw group was located at the bottom of the tibial plateau, whereas in the metal block extension stem group, the point of maximum stress was at the distal end of the extension stem (Figure 4).

#### 3.2.3 von Mises stress (VMS) distribution and results in the proximal tibial bone defect region

In the case of a 5-mm bone defect, the maximum stress within the cement-screw group was 25.3 MPa, whereas the maximum stress in the metal block extension stem group was 16.3 MPa, indicating a 35.6% reduction in stress. For a 10-mm bone defect, the maximum stress in the cement-screw group was 24.1 MPa, while the maximum stress in the metal block extension stem group was 18.8 MPa, representing a 22.0% decrease in stress. There was a significant reduction in stress in the proximal tibial bone defect area for the metal block extension stem group (Figure 4).

#### 3.2.4 The peaks of micromotion between the prosthesis and the Tibia

**FIGURE 5:**
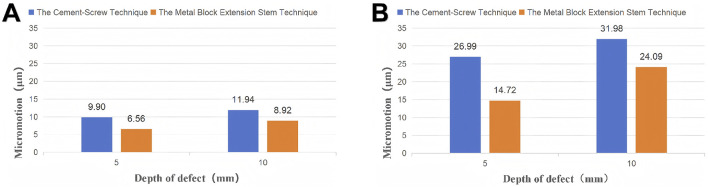
The peaks of micromotion between the prosthesis and bone tissue for 5 mm and 10 mm bone defects treated using the cement-screw and metal block extension stem technique. **(A)** Application of a horizontal walking load. **(B)** Application of a descending stairs load.

For 5-mm bone defects, the peaks of micromotion was 26.99 μm in the cement-screw group and 14.72 μm in the metal block extension stem group. For 10-mm bone defects, the peaks of micromotion was 31.98 μm in the cement-screw group and 24.09 μm in the metal block extension stem group (Figure 5).

## 4 Discussion

Total Knee Arthroplasty (TKA) is a well-established surgical approach for treating end-stage knee osteoarthritis. Its clinical application can significantly improve joint function and enhance patients’ quality of life (Liu et al., 2024). Bone defects, however, are a common issue in severe knee osteoarthritis and revision knee arthroplasty. Achieving stable reconstruction of the defective area is directly related to the stability and longevity of the joint implant. Therefore, the reconstruction of tibial plateau defects stands as a core clinical challenge in TKA, and its treatment methods continue to attract academic attention. In current biomechanical research, finite element analysis has become the preferred method for stress analysis due to its advantages in digital simulation. Cho et al. (2022) reported that implant design affects the load distribution in the peri-prosthetic tibia, while Liu et al. (2025) reported the influence of screw implantation angle on biomechanical outcomes in the treatment of TKA tibial defects using the cement-screw technique. Therefore, by constructing finite element models, this study enables researchers to perform multi-parameter repetitive tests while avoiding ethical constraints, thereby providing a reliable basis for selecting reconstruction methods for tibial defects in TKA.

Through VMS (von Mises Stress) and micromotion assessments, this study systematically compared the biomechanical performance of the cement-screw technique and the metal block extension stem technique under different bone defect depths. The results indicated that, under two types of loading and two defect depths, the tibial stress in the metal block extension stem group was consistently lower than that in the cement-screw group. Moreover, as the bone defect depth increased, the stress in the cement-screw group gradually rose. Specifically, during descending stairs, the maximum tibial stress in the 10 mm bone defect group using the cement-screw technique reached 151 MPa. According to previous literature (Wan et al., 2024), when the stress on bone tissue exceeds 150 MPa, fractures are prone to occur. In contrast, the metal block extension stem group exhibited an opposite stress trend. We attribute this outcome to the disparity in elastic modulus among the metal, bone cement, and bone (particularly the cortical bone bearing the primary stresses). According to Table 1, the elastic modulus of bone cement (3.0 GPa) lies between that of cancellous bone (0.7 GPa) and cortical bone (14.0 GPa), but it is still significantly lower than that of cortical bone. Conversely, the elastic modulus of metal (110 GPa) is significantly higher than that of cortical bone. Within multi - material structures, loads tend to propagate through paths of greater stiffness. Consequently, in the metal block extension stem group, the highly rigid metal block and extension handle bear a greater proportion of the load, leading to reduced stress on the surrounding bone (the stress shielding effect). Conversely, in the cement - screw group, the relatively lower stiffness of bone cement provides weaker load “diversion,” allowing greater stress transmission to the surrounding bone and resulting in elevated peak stresses in the tibia (Kreuzer et al., 2016; Conlisk et al., 2017). For 5 mm bone defects, the stress advantages between the two techniques were not significant. However, for 10 mm bone defects, we recommend the metal block extension stem technique based on the study results.

Regarding the VMS distribution and results of the prosthesis, the study demonstrated that adding a stem reduced the proximal stress on the prosthesis and transferred it distally, with stress concentration occurring at the end of the stem. This finding aligns with previous research by Xie et al. (2020). In this study, the maximum stress on all prosthetic models was 194 MPa, far below the yield strength of Ti6Al4V material (795 MPa) (Tumulu and Sarkar, 2018), indicating that prosthetic fracture is unlikely under the study’s loading conditions.

For the VMS in the proximal tibial bone defect area, the stress distribution trends were consistent under both types of loading, with the metal block extension stem group exhibiting significantly lower stress than the cement-screw group. This finding is consistent with previous research by Liu et al. (2020). According to Wolff’s Law, bone morphology and internal structure adaptively remodel in response to changes in stress direction and magnitude. When stress stimulation falls below the physiological threshold, progressive bone loss is triggered. Against this backdrop, the differences in elastic modulus between metal implants and host bone tissue can induce stress shielding effects, exacerbating local bone resorption and ultimately compromising prosthesis-bone interface stability (Liu et al., 2020; Bugbee et al., 1996; Completo et al., 2013; Wegner et al., 2019). This phenomenon is a recognized clinical issue and has been confirmed by numerous clinical studies (Bugler et al., 2015; Martin-Hernandez et al., 2017; Tsukada et al., 2013).

Aseptic loosening is one of the common complications following TKA, with excessive micromotion at the interface between the prosthetic implant and adjacent bone tissue being a primary factor in its occurrence (Wan et al., 2024; Berahmani et al., 2016). Therefore, this study constructed a cement prosthesis model and analyzed the micromotion between the prosthesis and tibia. According to the micromotion results, under the same loading and defect depth, the micromotion between the prosthesis and tibia in the metal block extension stem group was significantly lower than that in the cement-screw group. Additionally, as the defect depth increased, the micromotion values in both groups showed an increasing trend. This phenomenon suggests that as the defect depth increases, the stability of the prosthetic implant gradually declines. Although the micromotion results were small in all models in this study, caution is warranted regarding whether larger micromotion values may occur under loading conditions not examined in this study, potentially affecting prosthetic implant stability and leading to aseptic loosening.

In summary, the results of this study indicate that for 5 mm tibial bone defects, the biomechanical advantages of the two techniques are not significantly different, but the cement-screw technique is more cost-effective and quicker. For 10 mm tibial bone defects, the cement-screw technique increases the risk of fractures. Under both defect depths, the metal block extension stem technique produces stress shielding and requires the removal of some healthy bone. Based on the finite element analysis results, we recommend that clinicians consider the patient’s actual condition when selecting a treatment for 5 mm bone defects and prioritize the metal block extension stem technique for 10 mm tibial plateau bone defects.

The findings of this study reveal the biomechanical differences between the two techniques under different loading conditions and defect depths, providing clinicians with an important reference for selecting appropriate implant solutions. However, this study has several limitations: ① The finite element model is derived from the data of a single male volunteer with normal bone density, failing to reflect biological variations across different age groups, genders, body mass indices, and bone densities (particularly in osteoporosis). Consequently, it primarily provides qualitative comparative trends, and its generalizability requires further validation. ② The constructed medial tibial defect model encompassed only two depths—5 mm and 10 mm—and did not explore other variables such as defect shape or lateral tibial plateau defects. Consequently, the conclusions primarily apply to specific defect conditions. ③ The model simplifies bone and implant materials as isotropic elastic materials, differing from the anisotropic properties of actual bone tissue. While the overall trends hold reference value, results should be extrapolated with caution. ④ Load conditions only simulate two daily activities: horizontal walking and descending stairs, failing to cover other common movement scenarios such as squatting and jumping. All these factors require refinement and validation in subsequent studies through approaches such as parametric modelling, multi-scenario loading, and multi-factor analysis.

## Data Availability

The original contributions presented in the study are included in the article/supplementary material, further inquiries can be directed to the corresponding author.
